# The acute effect of exercise modality and nutrition manipulations on post-exercise resting energy expenditure and respiratory exchange ratio in women: a randomized trial

**DOI:** 10.1186/s40798-015-0010-3

**Published:** 2015-06-05

**Authors:** Hailee L Wingfield, Abbie E Smith-Ryan, Malia N Melvin, Erica J Roelofs, Eric T Trexler, Anthony C Hackney, Mark A Weaver, Eric D Ryan

**Affiliations:** 1 Department of Exercise and Sport Science, University of North Carolina, 209 Fetzer Hall, CB #8700, Chapel Hill, NC 27599-8700 USA; 2 Department of Nutrition - Gillings School of Global Public Health, University of North Carolina, Chapel Hill, NC USA; 3 Departments of Medicine and Biostatistics, University of North Carolina, Chapel Hill, NC USA

## Abstract

**Background:**

The purpose of this study was to examine the effect of exercise modality and pre-exercise carbohydrate (CHO) or protein (PRO) ingestion on post-exercise resting energy expenditure (REE) and respiratory exchange ratio (RER) in women.

**Methods:**

Twenty recreationally active women (mean ± SD; age 24.6 ± 3.9 years; height 164.4 ± 6.6 cm; weight 62.7 ± 6.6 kg) participated in this randomized, crossover, double-blind study. Each participant completed six exercise sessions, consisting of three exercise modalities: aerobic endurance exercise (AEE), high-intensity interval running (HIIT), and high-intensity resistance training (HIRT); and two acute nutritional interventions: CHO and PRO. Salivary samples were collected before each exercise session to determine estradiol-β-17 and before and after to quantify cortisol. Post-exercise REE and RER were analyzed via indirect calorimetry at the following: baseline, immediately post (IP), 30 minutes (30 min) post, and 60 minutes (60 min) post exercise. A mixed effects linear regression model, controlling for estradiol, was used to compare mean longitudinal changes in REE and RER.

**Results:**

On average, HIIT produced a greater REE than AEE and HIRT (*p* < 0.001) post exercise. Effects of AEE and HIRT were not significantly different for post-exercise REE (*p* = 0.1331). On average, HIIT produced lower RER compared to either AEE or HIRT after 30 min (*p* < 0.001 and *p* = 0.0169, respectively) and compared to AEE after 60 min (*p* = 0.0020). On average, pre-exercise PRO ingestion increased post-exercise REE (*p* = 0.0076) and decreased post-exercise RER (*p* < 0.0001) compared to pre-exercise CHO ingestion.

**Conclusion:**

HIIT resulted in the largest increase in REE and largest reduction in RER.

**Electronic supplementary material:**

The online version of this article (doi:10.1186/s40798-015-0010-3) contains supplementary material, which is available to authorized users.

## Key points

- HIIT elicited the largest increase in post-exercise energy expenditure.
- HIIT resulted in the largest reduction in post-exercise RER, increasing fat oxidation, compared to AEE and HIRT.
- In combination with varying exercise modalities, PRO intake elevated post-exercise REE and fat oxidation (via RER) to a greater extent than CHO.
- Integrating HIIT and pre-exercise PRO intake into exercise routines for women, may have positive implications on weight and body composition.

## Background

More than 60% of women in the United States are overweight, and 1/3 of those women are obese [1]. In addition, 75% of normal weight women believe they are overweight and 90% overestimate their body size [2]. With such high obesity and body dissatisfaction rates, it is important for women to receive reliable health and weight loss recommendations. In addition, lack of time has shifted the focus on more practical time-efficient strategies for exercise.

For health and weight maintenance, aerobic endurance exercise (AEE) is usually prescribed [3]. However, more recently, higher-intensity exercise modalities have been suggested as more time-efficient strategies for improvements in health and energy expenditure [4-6]. Post-exercise resting energy expenditure (REE) has been reported to increase following a 20-min treadmill run [4], while high-intensity interval training (HIIT) has increased total caloric energy expenditure (EE) in half the time [7]. In addition, high-intensity resistance training (HIRT) with short rest intervals has been shown to enhance post-exercise REE above that seen with more commonly prescribed resistance training [8] of lesser intensity and longer rest periods. While these three common exercise modes seem to be successful at expending calories, previous research has indicated that high-intensity exercise stimulates similar increases in post-exercise REE in comparison to lower intensity exercise, in half the time [9,10]. Research is still conflicted on whether aerobic or resistance exercise is more effective for augmenting EE. Higher intensity exercise has also been linked to higher rates of fat oxidation, measured by respiratory exchange ratio (RER) [6,11,12].

The combined effect of varying exercise modalities and pre-workout nutrition on energy substrate utilization and EE in women is limited. Pre-exercise ingestion of protein (PRO) has been found to increase post-exercise REE more than that of pre-exercise ingestion of carbohydrate (CHO) [13,14]. While women rely heavily on fat as an energy substrate during exercise, previous data has demonstrated the importance of pre-exercise feedings on augmenting lipolysis [15]. In addition, a higher rate of fat oxidation in women than men in the fasted state, but not in the postprandial state, has been reported [16]. Pre-exercise nutrient timing and content may be important when evaluating exercise substrate utilization in women.

When evaluating exercise and nutritional responses in women, it is important to consider hormonal variances, such as the sex-specific hormone estradiol that varies throughout the menstrual cycle. Estradiol has been shown to have an impact on REE [17-19]. Controlling for estradiol concentrations in metabolic evaluations is crucial for determining the practical effects an intervention may have [18]. High-intensity aerobic exercise has been shown to stimulate increases in cortisol [20], a stress hormone that regulates energy substrate utilization by mobilizing amino acids from skeletal muscle and promoting gluconeogenic activity [21]. Elevated cortisol concentrations have been reported as a primary hormonal factor augmenting exercise adaptations from high-intensity exercise, as well as impacting rates of lipolysis [20].

To date, no investigations have evaluated the combined effects of varying exercise modalities and acute nutritional supplementation in women. Therefore, the purpose of the current study was to examine the effect of common exercise modalities, AEE, HIIT, and HIRT, combined with pre-exercise CHO or PRO ingestion, on post-exercise REE and RER in women.

## Methods

### Experimental design

To test the study hypotheses, a randomized, crossover, factorial design was used. After preliminary resting heart rate, body composition, and strength testing, each participant completed six randomly ordered exercise sessions consisting of three exercise modalities: AEE, HIIT, and HIRT; and two acute randomized nutritional interventions: CHO and PRO (Figure 1). Participants performed each exercise mode twice, with a different nutritional intervention each time. Salivary samples were collected before each exercise session to determine estradiol and cortisol concentrations and after each exercise session to determine cortisol concentrations. Exercise sessions were performed at the same time of day, within 2 h, with at least 48 h between each session, and the order was randomly assigned using random allocation software (version 1.0.0; Isfahan, Iran). Subjects were instructed to stay well hydrated, to be 3 h postprandial, to refrain from caffeine 5 h prior, and to refrain from strenuous exercise 24 h prior to all exercise sessions.

**Figure 1:**
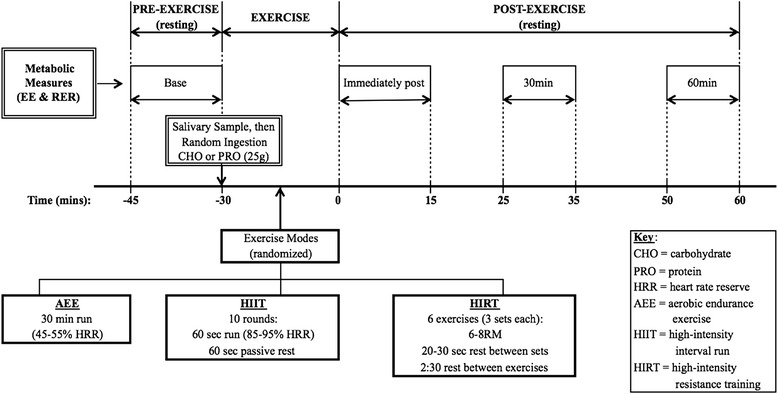
**Experimental protocol schematic.** REE, resting energy expenditure (kcal/day); RER, respiratory exchange ratio; CHO, carbohydrate; PRO, protein; HRR, heart rate reserve; AEE, aerobic endurance exercise; HIIT, high-intensity interval run; HIRT, high-intensity resistance training.

### Participants

Twenty-one eumenorrheic, college-aged, recreationally active women were enrolled to participate in this study; one participant withdrew due to unrelated injury. Twenty women (*n* = 20) completed the study (Table 1) and were included in the statistical analyses. The study protocol was approved by the University’s Institutional Review Board, and all procedures followed were in accordance with the Helsinki Declaration of 1975, as revised in 2008. Prior to participation, all participants provided written informed consent and completed a health history questionnaire. To be included in this study, participants had to be a woman between the ages of 18 and 35 and be recreationally active, defined as accumulating 1 to 5 h per week of aerobic and/or resistance exercise, excluding competitive athletes. Participants were excluded if they were unfit, pregnant, had any cardiovascular or neuromuscular health risks, had injuries, or had any heart, lung, kidney, or liver disease.

**Table 1:** **Descriptive statistics for all subjects (** ***n*** **= 20) at baseline**

	**Mean ± SD**
Age (years)	24.6 ± 3.9
Height (cm)	164.4 ± 6.6
Weight (kg)	62.7 ± 6.6
Fat mass (kg)	17.6 ± 4.0
Lean mass (kg)	42.5 ± 4.4
Percent body fat (%)	28.2 ± 4.8

### Heart rate reserve measurement and body composition assessment

Participants reported to the Applied Physiology Laboratory after an 8-h fast, where they rested for 15 min. Resting heart rate (RHR) was measured using a polar heart rate monitor (Polar FT1, Polar USA, Port Washington, NY, USA), while age-predicted maximal heart rate (MHR) was determined. Exercise target heart rate (THR) was calculated using the Karvonen equation [THR = ((MHR − RHR) % intensity) + MHR] [3]. During each exercise bout, heart rate (HR) was measured using the aforementioned HR monitor. Exercises were matched for caloric expenditure (pilot data not published), opposed to time, in order to control for exercise length.

For baseline data, whole body composition was measured using a Hologic Dual Energy X-ray Absorptiometer (DEXA, Hologic Discovery W, Bedford, MA, USA) using the device’s default software (Apex software version 3.3). The device uses rectilinear fan beam acquisition to give a three-compartment assessment of body composition, including fat mass (FM), lean mass (LM), and percent body fat (% fat). After removing all metal objects, subjects laid supine in the middle of the platform with hands facedown near their sides. Subjects were instructed to remain still and breathe normally for the duration of the scan. All scans were performed by the same DEXA-certified technician. The device was calibrated according to the manufacturer recommendations before testing to ensure valid results. Previous test-retest reliability in our lab were FM: ICC = 0.98, SEM = 0.85 kg; LM: ICC = 0.99, SEM = 1.07 kg; % fat: ICC = 0.98, SEM = 1.0%.

### Maximal strength test

In a standard post-absorptive state, each participant performed a one-repetition maximum (RM) strength test for bench press and leg press, using free weights and a spotter, according to standard guidelines previously used in this laboratory. After a 5-min warm-up and light stretching, participants were familiarized with the equipment and the motion of the movements. Each participant then performed eight to ten repetitions at 50% of their predicted 1RM. After a 1-min rest period, each participant performed four to six repetitions at 80% of their predicted 1RM. Following a 1-min rest period, the weight was increased to an estimated 1RM load, which participants lifted one time. After each successful set of one repetition, the weight was increased until a failed attempt occurred, within four attempts. Two to three minutes of rest was given between 1RM attempts.

After the 1RM tests, participants performed a multiple-RM strength test for four accessory exercises: alternating stationary lunge, overhead shoulder press, biceps curls, and overhead triceps extension. With free weights and a spotter, participants performed each exercise with a weight they could lift for one set of no less than three, and no more than ten repetitions. The weight for each exercise, set by the lab assistants, was based on each participant’s past strength training experience. If the participant was unable to perform the exercise for three repetitions or was able to perform more than ten repetitions, the weight was decreased or increased, respectively, and the test was redone. A 2- to 3-min rest was given between each exercise.

The multiple RM obtained from the strength tests was used to estimate 1RM for the accessory exercises using the following equation [22]:

$$ 1\mathrm{R}\mathrm{M}=\frac{\mathrm{RepWt}}{0.522+{0.419}^{\left(-0.055 \times \mathrm{R}\mathrm{T}\mathrm{F}\right)}} $$

where RepWt = amount of weight lifted (lbs) with each repetition and RTF = amount of repetitions to fatigue.

Once the participant’s 1RM was determined for all exercises, 80% to 85% was calculated for the 6RM to 8RM used for the HIRT sessions.

### Energy intake assessment

All participants completed a baseline 3-day food record to assess their regular nutrient intake. Participants were educated on food portions and were asked to eat similar diets the day prior to each exercise session to facilitate similar macronutrient profiles. Nutrition intake was analyzed using a nutrition software program (The Food Processor, version 10.12.0, Esha Research, Salem, OR, USA). On average, subjects ingested 2,078.7 ± 679.9 kcal, 253.7 ± 97.6 g CHO (approximately 48.8% CHO), 84.3 ± 29.9 g PRO (approximately 16.2% PRO), and 80.9 ± 36.7 g of fat (approximately 35.0% fat) per day.

### Saliva collection and analysis

In order to account for possible energy substrate utilization differences between the exercise sessions, estradiol was measured. Estradiol concentrations were determined by a 2.5 to 5.0 mL saliva sample prior to each exercise bout, using an ELISA assay for salivary estradiol-β-17 (estrogen) (Salivary 17β-Estradiol Enzyme Immunoassay Kit**,** Salimetrics, LLC, State College, PA, USA). To ensure valid saliva collection results, participants were asked to avoid drinking alcohol for 12 h and eating a major meal for 3 h prior to giving saliva samples. Participants were asked to rinse their mouth with water 10 min prior to saliva collection, to remove food residue. To avoid blood in saliva collections, participants were asked to avoid brushing teeth for 45 min and obtaining dental work for 48 h prior to giving saliva samples. All samples were maintained at 4°C no longer than necessary before freezing them at −20°C. Intra-assay precision coefficient of variation (CV) for estrogen was 8.7% to 18.6%; inter-assay precision CV was 3.9%. Due to the physiological role of estradiol-β-17 on energy substrate utilization in women [17,19], baseline estradiol levels were used as covariates in the REE and RER analyses.

Cortisol was measured to account for the stress response from each modality of exercise. Cortisol concentrations were determined by a 2.5 to 5.0 mL saliva sample prior to and following each exercise bout, using an ELISA Assay for salivary cortisol (Salivary Cortisol Enzyme Immunoassay Kit**,** Salimetrics, LLC, State College, PA, USA); the aforementioned protocol for collection and storage was used. Once all samples were collected, smaller subsamples were pooled between the two supplements to produce one pre- and one post-exercise sample to determine the effect of each modality on cortisol, independent of treatment. Intra-assay precision CV for cortisol was 12.0% to 17.1%; inter-assay precision CV was 4.9%.

### Nutritional intervention

After providing a saliva sample and immediately prior to beginning each exercise session, in a double-blind fashion, participants orally ingested 25 g of CHO (maltodextrin) or PRO (whey isolate; Elite Whey Protein Isolate, Dymatize Nutrition, Farmers Branch, TX, USA) mixed with 6 oz of water in an opaque bottle. Treatment order was randomly assigned using random allocation software. In accordance to the CONSORT guidelines, nutritional products were blinded by the company prior to arrival to the laboratory. All participants and research team members were blinded to the treatment, until after the statistical analyses.

### Aerobic endurance exercise (AEE)

An AEE bout consisted of a self-selected 5-min warm-up, followed by a 30-min treadmill (Q65 Series 90, Quinton Instrument Co., Seattle, WA, USA) jog at 45% to 55% HRR [4]. RPE (Borg scale) was recorded, and HR was measured at the end of each minute; both were averaged over the 30-min exercise period.

### High-intensity interval running (HIIT)

A HIIT bout consisted of a self-selected 5-min warm-up, followed by ten rounds of a 60-s treadmill run at 85% to 95% HRR with a 60-s passive rest period. The entire exercise bout lasted approximately 20 min. RPE was recorded, and HR was measured at the end of each interval; and an average of the ten 60-s exercise intervals was used for statistical analysis.

### High-intensity resistance training (HIRT)

Data obtained from the strength assessment were used to determine an appropriate weight load for the HIRT session. Exercises were performed in the following order: leg press and bench press (York Barbell Co., York, PA, USA), lunges, shoulder press, biceps curl, and triceps extension using free weights. Subjects performed a self-selected warm-up prior to starting. A HIRT bout consisted of three sets of 6RM to 8RM followed by a 20- to 30-s rest for each exercise. There was a rest period of 2.5 min between each exercise [8]. The average length of HIRT sessions was approximately 25 min. HR was measured at the end of each set, and RPE was recorded at the end of each exercise; the average of HR and RPE of each of the six exercises was used in the statistical analyses.

### Metabolic measurements

REE and RER were analyzed using a metabolic cart and internal software (TrueOne 2400, ParvoMedics, Inc., Sandy, UT, USA). Indirect assessments of oxygen uptake (VO_2_) and carbon dioxide production (VCO_2_) were measured and used in the following equations to calculate REE [23] and RER [24], respectively:

$$ \mathrm{R}\mathrm{E}\mathrm{E}\ \left(\mathrm{kcal}/\mathrm{day}\right)=\left[\left(3.9*\left({\mathrm{VO}}_2\ \left(L*{ \min}^{-1}\right)\left)\right)+\left(1.1*\right({\mathrm{VCO}}_2\ \left(L*{ \min}^{-1}\right)\right)\right)\right]*1440\kern0.5em  \min $$

$$ \mathrm{R}\mathrm{E}\mathrm{R}=\frac{{\mathrm{VCO}}_2\ \left(L*{ \min}^{-1}\right)}{{\mathrm{VO}}_2\ \left(L*{ \min}^{-1}\right)} $$

The gas analysis was performed via a mouthpiece and hose immediately prior to each exercise session, for 15 min, to obtain resting measures (base). Immediately after the conclusion of the exercise sessions, participants were seated and reconnected to the metabolic cart for 15 min to obtain immediately post- (IP) exercise measures. The participants were then disconnected from the cart and remained quietly seated. Measurements were taken again during minutes 25 to 35 (30 min) and 50 to 60 (60 min).

### Statistical analyses

A one-way repeated measures ANOVA was performed to determine baseline differences in salivary estradiol and cortisol levels. An analysis of covariance (ANCOVA) mixed effects linear model was used to compare mean longitudinal changes in REE and RER. Initial models included fixed effects for nutritional treatment (CHO vs. PRO), exercise modality (AEE vs. HIIT vs. HIRT), time (baseline vs. IP vs. 30 min vs. 60 min), two-way and three-way interactions, as well as baseline salivary estradiol level. The three-way interaction was tested first; if non-significant, a reduced model was fit without the three-way interaction. Multiple degree of freedom contrasts were then used to test for any evidence of differences between nutritional interventions or exercise modalities over time. Only if those overall contrasts were significant, pairwise comparisons were completed. An ANCOVA was performed on the change in cortisol, covaried for baseline differences. A repeated measures ANOVA was performed on HR and RPE differences in modalities. SPSS version 20 (IBM; Armonk, NY, USA) was used to perform the statistical analyses. All tests were conducted at the 5% significance level.

## Results

### Estradiol

There was no significant difference (*p* = 0.636; ES = 0.035) between baseline estradiol concentrations for each exercise session. While this *p* value was not significant, the concentrations obtained were physiologically different between and within subjects. Concentrations spanned from 0.00 to 5.04 pg/mL between subjects, with one subject ranging 0 to 4.70 pg/mL between testing days.

### REE (kcal/day)

The three-way interaction for REE was non-significant (*p* = 0.634), so it was removed from the model. The modality and treatment interaction was not significant (*p* = 0.060) (Additional file 1: Appendix 1), so main effects over time are reported. Significant two-way interactions were found between modality and time (*p* < 0.001) and time and treatment (*p* = 0.008), indicating differential changes over time.

Significant modality differences were found between HIIT and AEE (*p* < 0.001), and HIIT and HIRT (*p* < 0.001) over time, but not between AEE and HIRT (*p* = 0.133). Mean REE significantly increased more with HIIT compared to AEE from baseline to IP exercise (*p* < 0.001), 30 min post (*p* = 0.002), and 60 min post (*p* = 0.002); (Additional file 1: Appendix 1 and Figure 2A). Mean REE significantly increased more with HIIT than HIRT from baseline to IP exercise (*p* < 0.001) but was not significantly different at 30 min (*p* = 0.335) or 60 min (*p* = 0.143). Mean REE significantly increased more following PRO compared to CHO IP (*p* = 0.007), 30 min (*p* = 0.010), and 60 min (*p* = 0.002) (Figure 2B).

**Figure 2:**
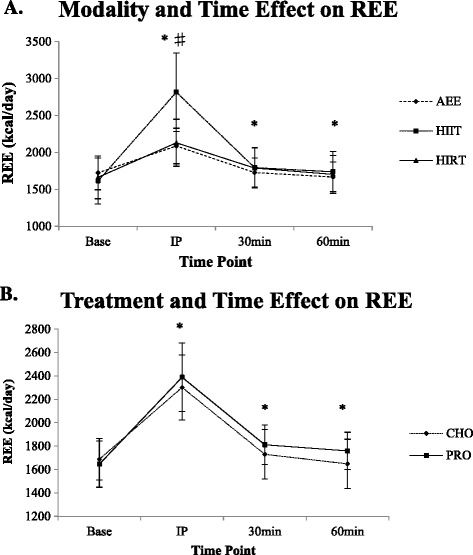
**Energy expenditure (REE; kcal/day) as a result of A) modality and time and B) treatment and time.** Values expressed as mean ± SD. **A)** Asterisk indicates significant difference between AEE and HIIT (*p* < 0.001 to *p* = 0.002); Number sign indicates significant difference between HIIT and HIRT (*p* < 0.001). **B)** Asterisk indicates significant difference between CHO and PRO (*p* = 0.002 to *p* = 0.010). REE, resting energy expenditure (kcal/day); CHO, carbohydrate; PRO, protein; AEE, aerobic endurance exercise; HIIT, high-intensity interval run; HIRT, high-intensity resistance training; Base, baseline measurement; IP, immediately post-exercise measurement; 30 min, 30 minutes post-exercise measurement; 60 min, 60 minutes post-exercise measurement.

### RER

The three-way interaction for RER was non-significant (*p* = 0.161), so it was removed from the model. The interaction between modality and treatment was not significant (*p* = 0.650), so main effects were over time. Significant two-way interactions were found between modality and time (*p* < 0.001) and time and treatment (*p* < 0.001), indicating differential changes over time.

Significant over-time differences were found between HIIT and AEE (*p* < 0.001), HIIT and HIRT (*p* < 0.001), and AEE and HIRT (*p* = 0.002). As a result of HIIT, mean RER increased significantly more than AEE and HIRT from baseline to IP exercise (*p* < 0.001) (Additional file 1: Appendix 2 and Figure 3A). However, mean RER significantly decreased more at 30 (*p* < 0.001) and 60 min post exercise (*p* = 0.002) for HIIT than AEE. Mean RER significantly reduced more from baseline to 30 min (*p* = 0.017) but was not significantly different at 60 min post (*p* = 0.360) for HIIT when compared to HIRT. When comparing HIRT and AEE, there was no significant difference from baseline to IP exercise (*p* = 0.337), but mean RER was significantly lower for HIRT to 30 (*p* < 0.001) and 60 min (*p* = 0.027). Mean RER was significantly different over time between CHO and PRO IP exercise (*p* < 0.001) and was significantly lower for PRO compared to CHO from baseline to 30 (*p* = 0.001) and 60 min post (*p* < 0.001) (Figure 3B).

**Figure 3:**
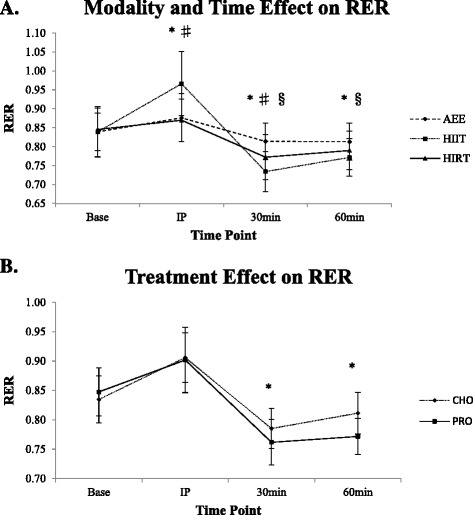
**Respiratory exchange ratio as a result of A) modality and time and B) treatment and time.** Values expressed as mean ± SD. **A)** Asterisk indicates significant difference between AEE and HIIT (*p* < 0.001 to *p* = 0.002); Number sign indicates significant difference between HIIT and HIRT (*p* < 0.001 to *p* = 0.017); Section sign indicates significant difference between AEE and HIRT (*p* = 0.001 to 0.027). **B)** Asterisk indicates significant difference between CHO and PRO (*p* < 0.001 to *p* = 0.001). RER, respiratory exchange ratio; CHO, carbohydrate; PRO, protein; AEE, aerobic endurance exercise; HIIT, high-intensity interval run; HIRT, high-intensity resistance training; Base, baseline measurement; IP, immediately post-exercise measurement; 30 min, 30 minutes post-exercise measurement; 60 min, 60 minutes post-exercise measurement.

### Cortisol

ANCOVA change scores demonstrated no significant effect of exercise modality on cortisol values (*p* = 0.168; ES = 0.015) (Table 2).

**Table 2:** **Change in cortisol (μg/dL) as a result of modality (mean ± SD)**

**Modality**	**Base**	**Post**	**∆**
AEE	0.39 ± 0.31	0.39 ± 0.33	0.00 ± 0.22
HIIT	0.44 ± 0.40	0.59 ± 0.50	0.15 ± 0.23
HIRT	0.35 ± 0.18	0.40 ± 0.20	0.05 ± 0.14

AEE, aerobic endurance exercise; HIIT, high-intensity interval run; HIRT, high-intensity resistance training; Base, baseline measurement; Post, post-exercise measurement; ∆, change in cortisol (μg/dL).

### HR and RPE

There was a modality effect on average HR (*p* < 0.001) and RPE (*p* < 0.001). Compared to HIIT, AEE produced a significantly lower mean HR (*Δ* = −58 bpm; *p* < 0.001) and RPE (*Δ* = −6; *p* < 0.001) (Table 3). There was no difference in HR (*Δ* = −3 bpm; *p* = 0.834) between AEE and HIRT. Mean RPE was significantly lower for AEE compared to HIRT (*Δ* = −5; *p* < 0.001). HIIT resulted in significantly higher mean HR (Δ = 54 bpm; *p* < 0.001) and RPE (*Δ* = 1; *p* = 0.001) compared to HIRT.

**Table 3:** **Average HR and RPE during the duration of each modality (Mean ± SD)**

	**HR (bpm)**	**RPE**
AEE	126 ± 7*	10 ± 1*^§^
HIIT	184 ± 29^#^	16 ± 1^#^
HIRT	129 ± 17	15 ± 1

AEE, aerobic endurance exercise; HIIT, high-intensity interval run; HIRT, high-intensity resistance training; HR, heart rate (bpm); RPE, rating of perceived exertion (Borg scale).

^*^Indicates significant difference between AEE and HIIT (*p* < 0.0001).

^#^Indicates significant difference between HIIT and HIRT (*p* < 0.0001 to *p* = 0.001).

^§^Indicates significant difference between AEE and HIRT (*p* < 0.0001).

## Discussion

Previous research evaluating the effect of AEE, HIIT, and HIRT individually on energy expenditure and energy substrate utilization have failed to provide direct comparisons between exercise modes; as well as failing to evaluate these effects in women. The current study is the first to compare common exercise modalities, with acute nutritional intake, on post-exercise REE and RER in women. Results of the current study indicate that HIIT produces a significantly higher REE than AEE up to 60 min post exercise and a significantly higher post-exercise REE than HIRT IP exercise; while AEE and HIRT produced similar post-exercise REE responses. HIIT and HIRT both also stimulated greater fat utilization (via a lower RER) when compared to AEE. In the current study, ingesting PRO prior to exercise produced an increased post-exercise resting energy expenditure, compared to CHO consumption. PRO ingestion also resulted in significantly augmented fat utilization 30 and 60 min post exercise.

### REE

As hypothesized, a single bout of HIIT produced a significantly higher post-exercise REE than AEE through 60 min post exercise (*Δ* = 189.2 kcal/day). Similar to the current study, higher post-exercise net EE with high-intensity short-duration cycling (75% VO_2max_) compared to shorter and longer durations at a low intensity (50% VO_2max_) has been found in men [9], indicating that exercise intensity affects the magnitude and duration of excess post-exercise oxygen consumption (EPOC). HIIT’s metabolic inefficiency has been contributed to rapid changes in skeletal muscle oxidative capacity due to the high level of muscle fiber recruitment, particularly in type II fibers [25]. In contrast to the current study, bouts of 60 s of work with 60 s rest, at 90% VO_2max_, have been found to have the same total net EE as 20-min bouts at 70% VO_2max_ [7]. Perhaps the difference in exercise intensities for the two sessions was not substantial enough to elicit significant differences, such as those found in the current study. Additionally, it is unclear if differences between exercise intensities were controlled for [7]. Subjects in the present study completed 30 min, instead of 20 min, of AEE in order to closely match EE during HIIT (determined during pilot testing; unpublished data).

In contrast to our hypothesis, when compared to HIIT, REE from HIRT was significantly lower from baseline to IP exercise (*Δ* = −169.3 kcal/day), with no differences at 30 and 60 min post exercise. Additionally, there was no difference between AEE and HIRT (*Δ* = 19.8 kcal/day). Research that has examined REE differences between AEE or HIIT and HIRT is limited. However, single bouts of resistance training and running performed at the same intensity have both resulted in elevated REE up to 10 h post exercise [26]. Contrary to the current study, total body circuit weight training (three sets of 15 repetitions at 65% 1RM) has been shown to elicit a higher post-exercise REE in women than a treadmill run matched for aerobic energy cost [27]. The current study also matched EE demands of AEE and HIRT during pilot testing, with HIRT being slightly lower, yet at a higher intensity than that of Braun et al. [27]. Post-exercise REE data from the current study eludes to exercise intensity, instead of duration, as the primary determinant of EPOC variance, similar to several studies [4-6]. Collectively, it seems as if HIIT is the most time-effective exercise modality for increasing EE in women.

Combined acute PRO feedings with exercise resulted in a significantly higher REE for PRO than for CHO up to 60 min post exercise (*Δ* = 59.3 kcal/day). This is similar to previous findings [13,14] that demonstrate greater post-exercise REE and fat oxidation following a pre-exercise meal or snack with higher PRO content. The increase in post-exercise REE with PRO ingestion can be contributed to PRO’s thermic effect of food, which is higher and more prolonged than that of CHO and fat [28]. In women, higher PRO diets have elicited an increased effect on diet-induced EE, in comparison to a lower PRO diet [28,29], which may be explained by amino acid absorption and disposal costs [29]. Consuming an additional 100 kcal of PRO prior to exercise, as done in the current study, may increase EE up to an hour after exercise.

### RER

Exercise intensity has previously been shown to be a driving factor for energy substrate utilization during and after exercise. Immediately after HIIT, RER was significantly increased compared to AEE, indicating a higher utilization of CHO during and immediately after exercise. Within 25 min afterward, RER was significantly lower than AEE (*p* < 0.001), demonstrating higher fat utilization, which was maintained up to 60 min post. Similar to the current study, previous studies have reported a significantly lower RER post exercise with repeated bouts of moderate intensity cycling, compared to a single bout [12] and a lower RER with a lower vs. higher intensity cycle bout (50% and 70% VO_2max_) through 3 h post exercise, indicating increased fat utilization [11]. Previous data demonstrates differing exercise intensity can augment post-exercise REE and fat oxidation, but intervals do not produce differences compared to continuous exercise if total EE, duration, and intensity between the two remain the same throughout the exercise bout. In contrast to the current study, exercise bouts at 45% and 65% VO_2max_ resulted in similar RER values when EE of exercise was matched [30]. Although diets were kept constant, residual effects of pre-exercise meals could cause similar RER values, since a fasting period of only 2 h was implemented prior to exercise.

During the post-exercise period, oxygen consumption is elevated for a period of time as a result of homeostasis disruption caused by exercise. Physiological changes, such as those in cellular ion concentrations, tissue temperatures, and metabolite and hormone levels, take place into recovery, disabling oxygen consumption to lower back to resting levels [24]. This process of EPOC is related to exercise duration and intensity and can be influenced by sex-based hormones (estrogen) and macronutrient availability, among other things [24]. CHO is the primary fuel used during moderate- to high-intensity exercise; but post exercise, the body shifts from CHO to lipid energy sources, which lowers RER [30]. The current study demonstrates the fuel shift within 25 min post exercise, indicating greater post-exercise fat utilization with HIIT. When comparing HIIT and HIRT, RER was significantly higher IP, significantly lower at 30 min, but similar 60 min after exercise. RER was lower for HIRT than AEE at 30 (*p* < 0.001) and 60 min post exercise (*p* = 0.027). Little research has compared RER between aerobic and resistance training, but investigations that have been done present conflicting results. In contrast to this study, no post-exercise differences in RER were found between a 60-min circuit weight training bout (four sets of ten repetitions, 70% 1RM) and a cycling bout (70% VO_2max_) in men [31]. Similar to the current study, a lower RER was demonstrated 22 h after HIRT compared to more traditional resistance training (four sets of 8 to 12 repetitions, 1- to 2-min rest) [8]. In relation to sex, no differences were reported in post-circuit weight training RER between men and women [32]. It is important to examine the menstrual cycle due to the effects estrogen may have on energy substrate utilization, as the mid-luteal phase is characterized by high estrogen levels, which can enhance lipid utilization with exercise in women [19,33].

In the present study, no differences were found in RER between CHO and PRO IP exercise; but supporting our hypothesis, RER was significantly decreased for PRO compared to CHO at 30 min (*Δ* = 0.036; *p* = 0.001) and 60 min post exercise (*Δ* = 0.052; *p* < 0.001). Similarly, higher fat oxidation has been reported with a low CHO diet compared to a moderate CHO diet at 1 h post exercise [14]. Substrate oxidation can be influenced by substrate availability from dietary intake and physical activity. While this study only evaluated acute feedings, low CHO diets have been shown to decrease circulating insulin levels, which promotes fatty acid utilization in skeletal muscle [14], perhaps supporting the lower RER for PRO ingestion in the current study.

### Dietary intake

Chronic dietary manipulations have an impact on substrate utilization. Based on 3-day diet logs, participants consumed on average 48.8% CHO, 16.8% PRO, and 34.2% fat daily. Although intakes were within the Acceptable Macronutrient Distribution Ranges for Americans, average baseline PRO intake was 1.3 g∙kg^−1^ day^−1^ (0.5 g∙kg^−1^ day^−1^ higher than the Recommended Dietary Allowance) [34]. Interestingly, while average PRO intake was relatively high, an additional acute feeding of 25 g of PRO significantly augmented REE in the current study, perhaps supporting the suggestion that higher protein needs (1.63 g∙kg^−1^ day^−1^) may be necessary for women to maintain nitrogen balance [35]. While the importance of chronic dietary manipulations is apparent, the current study supports the benefit of acute PRO feedings on EE, in healthy women eating within dietary guidelines. Acute pre-exercise feedings have positively influenced fat oxidation, in comparison to a fasted state that blunted fat oxidation [16]. Future long-term studies identifying the acute effects on weight loss and body composition improvements in women would be valuable.

### Limitations

A limitation in the current study was the use of indirect calorimetry to measure REE and RER, rather than whole body direct calorimetry, which is considered the gold standard. Also, excluding the acute PRO and CHO supplementation, no dietary modifications were made, which could have a potential effect on the acute RER measurement. However, because no modifications were made, the study results may be more practical for a woman who only wants to change her pre-workout nutrition rather than her whole diet. In addition, since this was an acute intervention, and post-exercise measurements were only conducted for 60 min, it is difficult to predict longer term EE and energy substrate utilization. Specifically, EE calculations extrapolate our 60-min findings to a 24-h period (using Weir et al. [23] equation), potentially elevating the actual caloric differences (i.e. approximately 800 kcal). Future research should focus on determining the effects of chronic exercise and nutrition modifications in women using whole body calorimetry.

## Conclusion

This study indicates that HIIT elicited the largest increase in post-exercise REE, as well as augmented RER, compared to AEE and HIRT. Immediately post exercise, there was a heightened caloric effect from the HIIT bout stimulating approximately 800 kcal/day more than HIRT and AEE. Beginning around 30 min post exercise, HIIT and HIRT both had a higher fat utilization than AEE; at 60 min post, HIIT and HIRT resulted in similar energy substrate utilization in comparison to each other. In combination with varying exercise modalities, PRO intake elevated post-exercise REE and fat oxidation to a greater extent than CHO. PRO ingestion prior to exercise may help further maximize the caloric effect, with an additional approximately 90 kcal/day expended compared to CHO. Around 30 min post exercise, PRO increased fat utilization compared to CHO. Collectively, these findings suggest a potential benefit of integrating HIIT and pre-exercise PRO intake into exercise routines. Specifically in women, this strategy may have positive implications on their health, weight, and body composition.

## Additional file

Additional file 1:
**Appendix 1.** REE (kcal/day) for each modality and time (mean ± SD). **Appendix 2.** RER for each modality, treatment, and time (mean ± SD).
