# Supplementary material for: The acute effect of exercise modality and nutrition manipulations on post-exercise resting energy expenditure and respiratory exchange ratio in women: a randomized trial
Source: Sports Med Open. 2015 Jun 5;1:11. doi: 10.1186/s40798-015-0010-3 (PMC4512833; doi:10.1186/s40798-015-0010-3)
Supplement: Supplementary file 1 [file 40798_2015_10_MOESM1_ESM.pdf]

Appendix 1. REE (kcals/d) for each modality and time. (Mean ± SD).

Modality	Treatment	Base	IP	30min	60min
AEE	CHO	1746 ± 214	2026 ± 240	1649 ± 173	1573 ± 190
	PRO	1704 ± 247	2149 ± 230	1806 ± 193	1763 ± 175
	Mean	1725 ± 231	2088 ± 235	1728 ± 183	1668 ± 183
HIIT	CHO	1681 ± 312	2805 ± 469	1840 ± 279	1750 ± 281
	PRO	1546 ± 301	2828 ± 599	1750 ± 265	1728 ± 267
	Mean	1614 ± 307	2817 ± 534*♯	1795 ± 272*	1739 ± 274*
HIRT	CHO	1638 ± 281	2073 ± 340	1699 ± 311	1623 ± 294
	PRO	1688 ± 305	2189 ± 292	1879 ± 198	1786 ± 188
	Mean	1663 ± 293	2131 ± 316	1789 ± 255	1705 ± 241

* Indicates significant difference between AEE and HIIT (p<0.0001-p=0.0022)

♯ Indicates significant difference between HIIT and HIRT (p<0.0001)

REE = resting energy expenditure (kcal/day), AEE = aerobic endurance exercise, HIIT = high-intensity interval run, HIRT = high-intensity resistance training, CHO = carbohydrate, PRO = protein, Base = baseline measurement, IP = immediately post-exercise measurement, 30min = 30 minutes post-exercise measurement, 60min = 60 minutes post-exercise measurement

Appendix 2. RER for each modality, treatment, and time. (Mean ± SD)

Modality	Treatment	Base	IP	30min	60min
AEE	CHO	0.82 ± 0.05	0.88 ± 0.06	0.83 ± 0.05	0.83 ± 0.05
	PRO	0.86 ± 0.08	0.87 ± 0.06	0.80 ± 0.04	0.80 ± 0.04
	Collapsed	0.84 ± 0.07	0.88 ± 0.06	0.82 ± 0.05	0.82 ± 0.05
HIIT	CHO	0.83 ± 0.06	0.96 ± 0.07	0.74 ± 0.05	0.80 ± 0.05
	PRO	0.84 ± 0.04	0.97 ± 0.10	0.73 ± 0.05	0.75 ± 0.03
	Collapsed	0.84 ± 0.05	0.97 ± 0.09*♯	0.74 ± 0.05*♯	0.78 ± 0.04*
HIRT	CHO	0.85 ± 0.06	0.88 ± 0.05	0.78 ± 0.05	0.81 ± 0.04
	PRO	0.84 ± 0.05	0.86 ± 0.06	0.76 ± 0.07	0.77 ± 0.05
	Collapsed	0.85 ± 0.06	0.87 ± 0.06	0.77 ± 0.06§	0.79 ± 0.05§

* Indicates significant difference between AEE and HIIT (p<0.0001-p=0.0020)

♯ Indicates significant difference between HIIT and HIRT (p<0.0001-p=0.0169)

§ Indicates significant difference between AEE and HIRT (p=0.0004-0.0265) RER = respiratory exchange ratio, AEE = aerobic endurance exercise, HIIT = high-intensity interval run, HIRT = high-intensity resistance training, CHO = carbohydrate, PRO = protein, Base = baseline measurement, IP = immediately post-exercise measurement, 30min = 30 minutes post-exercise measurement, 60min = 60 minutes post-exercise measurement
